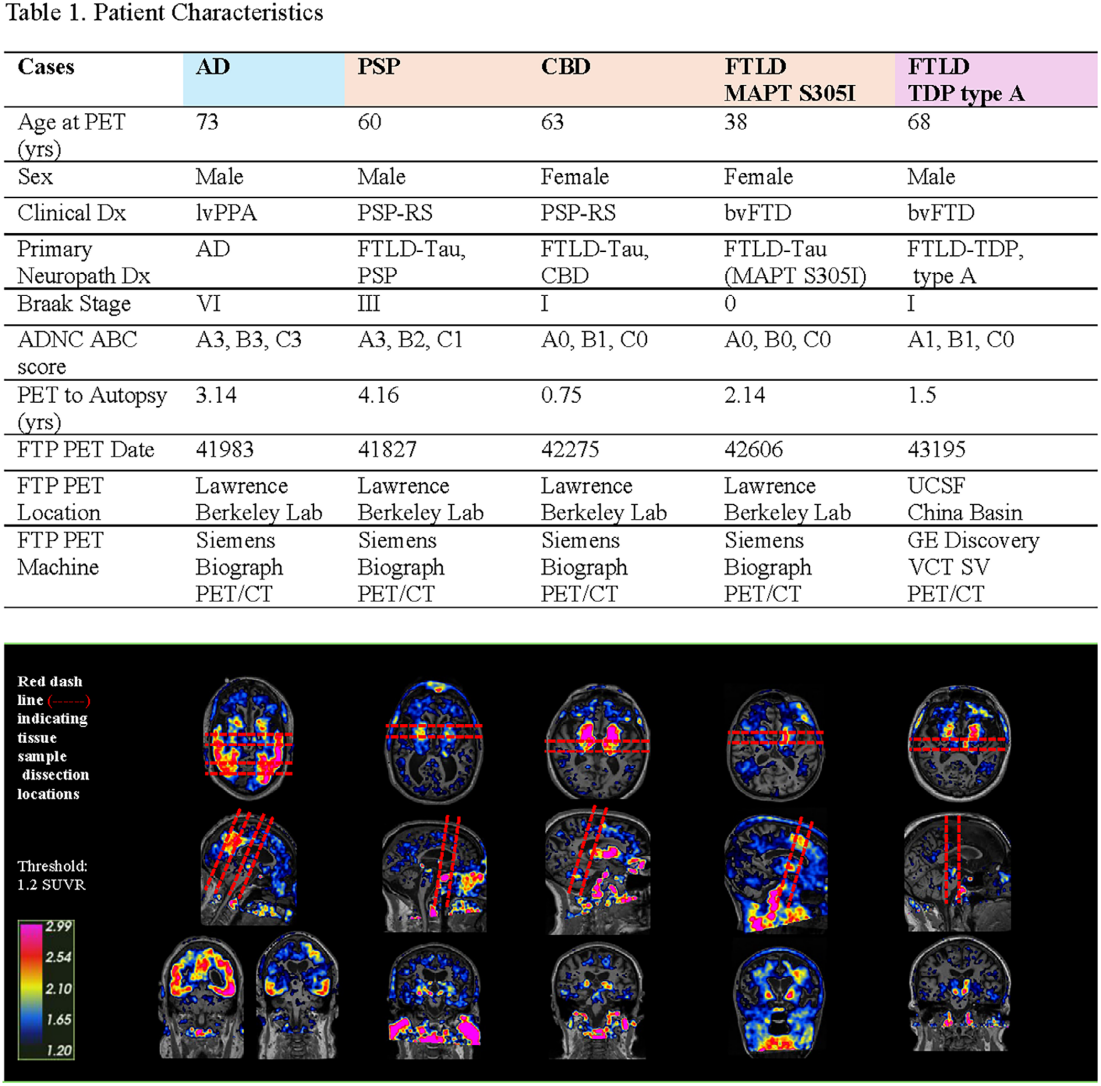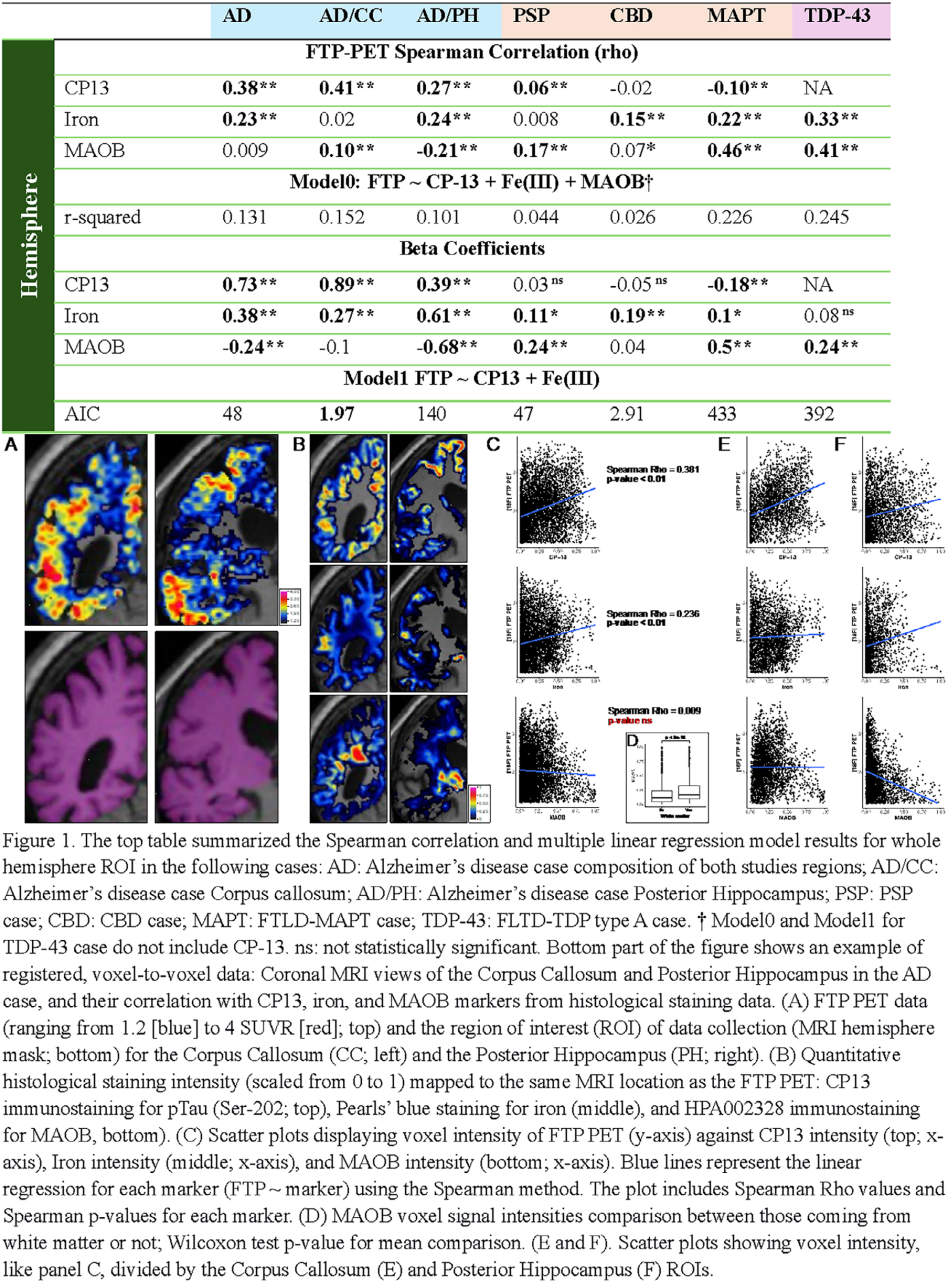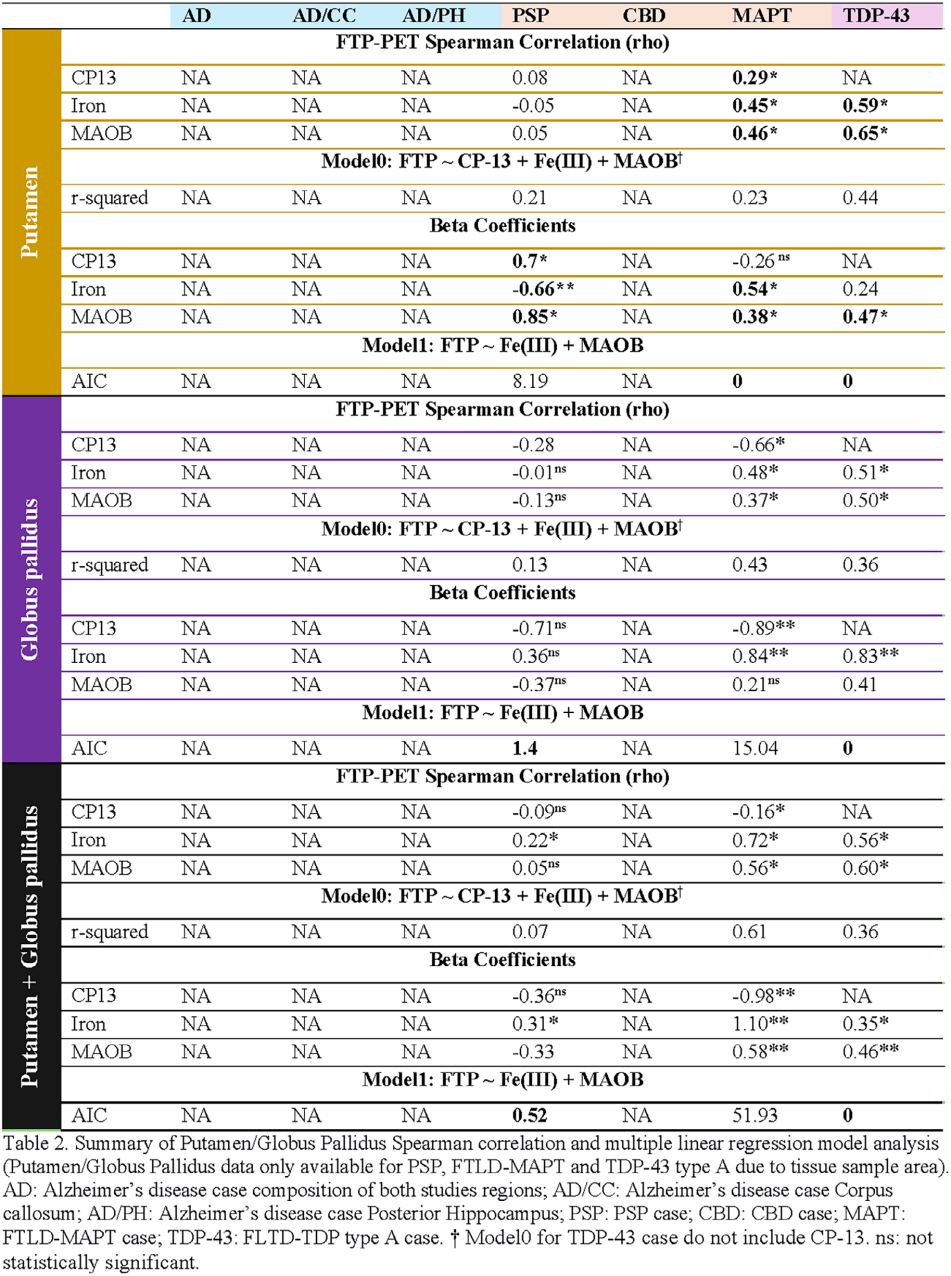# Unveiling the distinct roles of *p*‐tau, iron and MAOB in neurobiological basis of F18‐flortaucipir signal with voxel‐to‐voxel histology to PET analysis in different tauopathies

**DOI:** 10.1002/alz70856_105909

**Published:** 2026-01-09

**Authors:** Yuheng Chen, Renaud La Joie, Song Hua Li, Felipe Luiz Pereira, Lucile Zhu, Tia LaMore, Duygu Tosun, Daniela Ushizima, William W. Seeley, Salvatore Spina, Helmut Heinsen, Gil D. Rabinovici, Lea T. Grinberg

**Affiliations:** ^1^ Memory and Aging Center, UCSF Weill Institute for Neurosciences, University of California, San Francisco, San Francisco, CA, USA; ^2^ Memory and Aging Center, Weill Institute for Neurosciences, University of California San Francisco, San Francisco, CA, USA; ^3^ University of California, Berkeley, Berkeley, CA, USA; ^4^ Center for Imaging of Neurodegenerative Diseases, San Francisco Veterans Affairs Medical Center, San Francisco, CA, USA; ^5^ Lawrence Berkeley National Lab, Berkeley, CA, USA; ^6^ Department of Neurology, Memory and Aging Center, University of California San Francisco, San Francisco, CA, USA; ^7^ Julius‐Maximilians‐University Würzburg, Würzburg, Germany; ^8^ University of São Paulo, São Paulo, Brazil

## Abstract

**Background:**

Flortaucipir F^18^ was the first FDA approved PET tracer to detect tau pathology. Previous studies show that Flortaucipir is sensitive to Alzheimer's disease tau but not four‐repeat tauopathies. Furthermore, the neurobiological basis of low Flortaucipir signal remain unclear, especially in basal ganglia. In this study, we aimed to identify the contributions of *p*‐tau (Ser 202), iron/Fe(III), and MAOB to Flortaucipir signal in different tauopathies by performing voxel‐to‐voxel correlations between histological and PET‐CT volumes.

**Method:**

Coronal slides of ∼ 1 cm thickness from four tauopathy cases (AD, progressive supranuclear palsy, corticobasal degeneration, and (FTLD due to MAPT mutation P305S) and one FTLD‐TDP type‐A case were processed following a computational/histopathology pipeline (Ushizima, Chen, et al. 2021), allowing for whole 3D reconstruction of the histological maps at microscopical level, aligned to their corresponding MRI T1 maps (Figure 1). Serial 120 µm‐thick histological slides underwent immunohistochemistry to *p*‐tau (Ser 202, CP‐13) and MAOB, and Perl's iron staining. Density of pathological changes per voxel (1.22x1.22x120um^3^) was measured using convolution neural network algorithm. Flortaucipir images were thresholded at 1.2 SUVR. Spearman's bivariate and multiple linear regressions were used to compare Flortaucipir signal to histological counterparts (CP13/iron/MAOB) within whole hemisphere, putamen or globus pallidus.

**Result:**

Table 1 shows patient features. CP13(*p*‐tau) correlate the best to AD FTP‐PET, following with iron/Fe(III), then MAOB. FTP‐PET in non‐AD tauopathies correlate better to iron and MAOB with distinct patterns in putamen/globus gallidus: higher iron‐PET correlation in PSP and FTLD‐MAPT and higher MAOB‐PET correlation in TDP‐43 (Figure 1/Table 2) (DICE scores in whole hemisphere: 0.88 to 0.95 and putamen/globus pallidus: 0.76 to 0.90).

**Conclusion:**

Our findings strongly suggest that while FTP‐PET robustly reflects phospho‐tau pathology in AD, its interpretation in non‐AD tauopathies and TDP‐43 proteinopathies is more complex. Given the low affinity of FTP for the tau conformations present in non‐AD tauopathies, non‐tau substrates—particularly iron/Fe(III), and MAOB—are more likely to generate detectable signal than tau aggregates in such cases. The insights from our study highlight the importance of identifying additional off‐target contributors, refining tracer specificity, and integrating complementary markers to improve diagnostic accuracy and the utility of tau pet imaging in a broad range of neurodegenerative disorders.